# SRPK1 inhibition in prostate cancer: A novel anti-angiogenic treatment through modulation of VEGF alternative splicing

**DOI:** 10.1016/j.phrs.2016.03.013

**Published:** 2016-05

**Authors:** Athina Mavrou, Sebastian Oltean

**Affiliations:** a School of Physiology, Pharmacology and Neurosciences, UK; b School of Clinical Sciences/Bristol Renal, University of Bristol, UK

**Keywords:** Prostate cancer, Angiogenesis, Alternative splicing, Novel therapeutics

## Abstract

Prostate cancer remains one of the leading causes of cancer death in men around the world, regardless of intense research and development of novel therapies in the last 10 years. One of the new avenues that has been tested ⿿ inhibition of angiogenesis ⿿ has been disappointing so far in clinical studies in spite of strong evidence that determinants of angiogenesis (e.g. vascular endothelial growth factor) are strongly associated with disease progression. One of the reasons for these outcomes may be our poor understanding of the biology of angiogenesis in prostate cancer (and probably other cancers as well) resulting in inhibition of both detrimental and favourable molecules. We discuss here novel targeted and more specific approaches to inhibit angiogenesis in prostate cancer as well as a completely new therapeutic modality to do this ⿿ modulation of alternative splicing ⿿ that may be applicable to other molecules/biological processes as well.

## Graphical abstract

**Figure:**
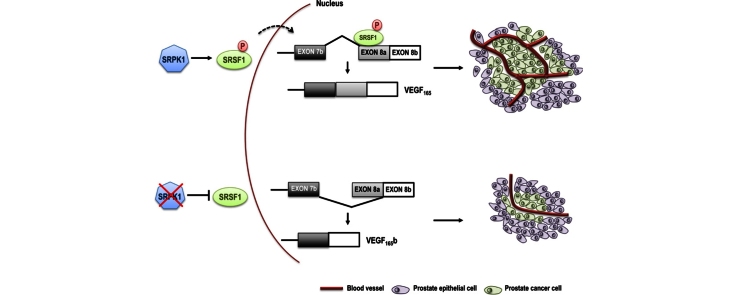

## 1 Introduction

In the last 15 years, there has been an increase in the use of drugs that target angiogenesis in cancers. The most well-known anti-angiogenic drug is Bevacizumab (Avastin), a humanized monoclonal antibody against vascular endothelial growth factor⿿A (VEGF-A) that is approved to be used in various cancers like colon cancer, non-small cell lung cancer or kidney cancer [1], [2]. However, following the initial excitement regarding the use of anti-angiogenics, they have not proven to induce a robust antitumoural treatment, with many clinical studies showing a modest progression-free survival and overall survival [3]. Additionally, side effects of such treatments may be quite important [1]. While there may be several explanations for this situation, it is more and more clear that we do not understand enough the vascular biology of tumours as well as many functional aspects of the molecules involved, therefore missing the chance to design more targeted treatments. This article discusses the current state of using anti-angiogenics in prostate cancer and our own work in finding a novel angle from which this problem may be solved.

## 2 Is there a rationale for developing anti-angiogenics in prostate cancer?

### 2.1 Prostate cancer

Prostate cancer (PCa) is the most commonly diagnosed cancer in men in the Western world. Given its incidence, PCa is one of the most considerable burdens on health systems around the world and accounts for a considerable number of death by cancer in men. The latest analysis of cancer statistics shows that in USA ⿼27,000 patients died of PCa in 2015 [4]. The mainstay of PCa therapy, aside from surgical intervention, is formed by a combination of anti-androgens, chemotherapy and radiation therapy. However, despite adequate treatment, a significant proportion of men progress to the metastatic castration-resistant form. It is therefore established that new avenues need to be found to be able to tackle this disease effectively. Indeed, in the last years several novel treatments involving immunomodulators, vaccines, epigenetic modifiers or bone-specific agents have been developed [5], [6], [7].

### 2.2 Evidence for the importance of angiogenesis in PCa progression

Induction of angiogenesis, the development of new vessels from existing ones, has long been recognized as an essential requirement for tumours to grow above a certain size and is therefore established as one of the hallmarks of cancer [8]. However, since angiogenesis should be more important in highly-vascularized cancers and not slow-growing like PCa it is essential to understand whether PCa progression is dependant on angiogenesis. In favour of this, several preclinical and clinical studies have shown a strong association of angiogenic factors with PCa. For example, VEGF, a main determinant of angiogenesis, was found to be increased in plasma and urine of patients with advanced PCa, whereas the microvessel density was strongly associated with Gleason score and metastasis [9], [10], [11].

### 2.3 Clinical trials using anti-angiogenics in PCa

Despite the above-mentioned evidence for the importance of angiogenesis in PCa, trials with different anti-angiogenic inhibitors combined with the main treatment for advanced PCa (Docetaxel and Prednisone) have failed to this date to show an improvement in overall survival [12]. There are several possible explanations for the failure of these trials, including hetereogeneity in patient stages and selection, treatment-related toxicities or activation of resistance mechanism through induction of pro-angiogenic factors [12], [13]. A recent phase II trial concludes that combination of anti-angiogenics (bevacizumab and lenalidomide⿿though arguably lenalidomide is not a ⿿pure⿿ anti-angiogenic) is able to circumvent the toxicity and may have clinical benefit [14]. Despite the insufficient data for the effectiveness of anti-angiogenic treatment in PCa, the above mentioned studies suggest that further research is required to establish the exact mechanism of regulation of angiogenesis in tumours. Are we inhibiting the right molecules? There is certainly room for improvement.

## 3 Alternative splicing and novel therapeutics

### 3.1 Alternative splicing as an important post-transcriptional regulation level

**Fig. 1:**
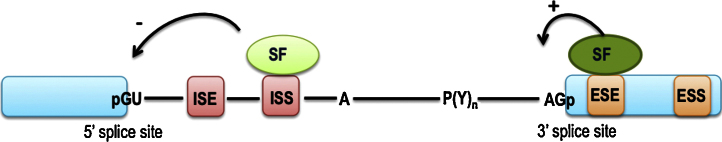
Basic mechanisms of splicing regulation. Positions of 5⿲ and 3⿲ splice sites, branch point (A) and polypyrimidine tract are shown. ESE and ESS: exon splicing enhancers and silencers respectively. ISE and ISS: intron splicing enhancers and silencers, respectively. Example of splicing regulation: binding of a splice factor (SF) to an ESE induces selection of a neighbouring splice site while binding to an ISI inhibits it.

Splicing is the removal of introns from the pre-mRNA and joining of exons to form the mature RNA. The splicing reaction is catalyzed by the spliceosome, a macromolecular ribonucleoprotein complex that assembles at splice sites (exon-intron junctions) and removes introns through two transesterification reactions. Beside splice sites (that have loose consensus sequences) the reactive sites in a basic splicing unit include a branch point involved in the transesterification reaction and a polypirimidine tract that binds crucial splice factors (Fig. 1).

**Fig. 2:**
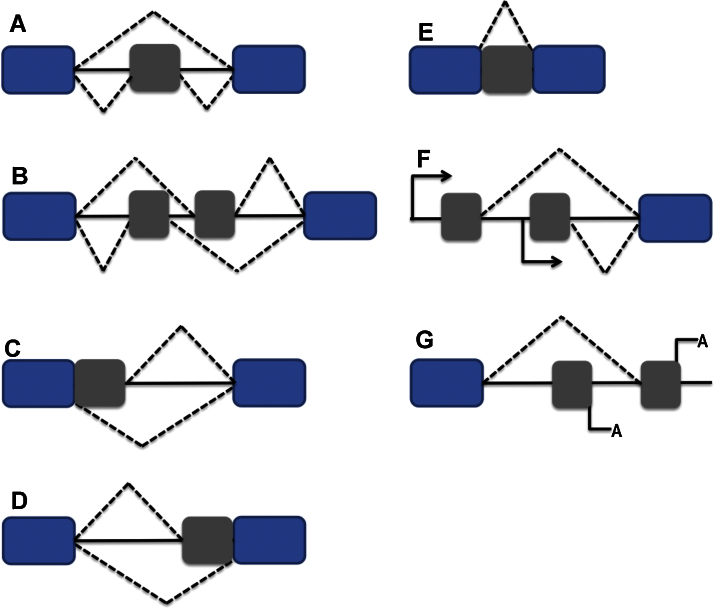
Common models of AS. (A) Cassette exon. (B) Mutually exclusive exon. (C) Alternative 5⿲ splice site. (D) Alternative 3⿲ splice site. (E) Intron retention. (F) Alternative promoter. (G) Alternative poly(A) site.

Alternative splicing (AS), the re-arrangement of exons, introns or parts of exons and introns in various combinations to result in multiple mature transcripts from the same pre-mRNA, has been described more than 40 years ago. There are several modes of AS with the main categories being: A) cassette exon ⿿ when an exon is either included or excluded in the mature transcript; B) mutually exclusive exons ⿿ a mature transcript contains either one of two exons but not both at the same time; C) and D) 5⿲ and 3⿲ alternative splice sites ⿿ resulting in inclusion/exclusion of parts of exons; E) intron retention ⿿ when an intron is not excised and appears in the mature RNA (Fig. 2). Combinations with other gene regulations levels may result in even more transcript diversity, e.g. F) alternative promoters or G) alternative poly(A) sites (Fig. 2)

What has only recently been established though, is how extensive AS is. Indeed deep sequencing studies have conclusively show that more than 95% of human genes are alternatively spliced [15], [16] providing a rationale for the existence of the estimated hundreds of thousands of proteins from only ⿼22,000 genes [17]. The extent of AS places this process as a major player in gene regulation and therefore determinant of cell function.

### 3.2 Regulation of AS

The decision to include or exclude a particular exon is based on the interaction between *cis*- and *trans*-acting *trans*-acting elements. *Cis* elements consist of regions were splice factors bind. Depending on the position and outcome of exon regulation they are divided in exonic and intronic splicing silencers (ESS and ISS) or exonic and intronic splicing enhancers (ESE and ISE) (Fig. 1).

Trans-acting regulatory molecules are splice factors ⿿ two of the most important classes that are ubiquitously expressed are serine-arginine (SR) proteins and heterogenous ribonucleoproteins (hnRNPs). A fair number of RNA-binding proteins have also been described to be splice factors and are not included in either of these two classes. Many of these have tissue-specific distribution or regulate defined processes like brain functions or muscle development ⿿ e.g. Nova, Rbm24 [18], [19] or the epithelial state of a cell ⿿ ESRP1 and 2 [20], [21].

**Fig. 3:**
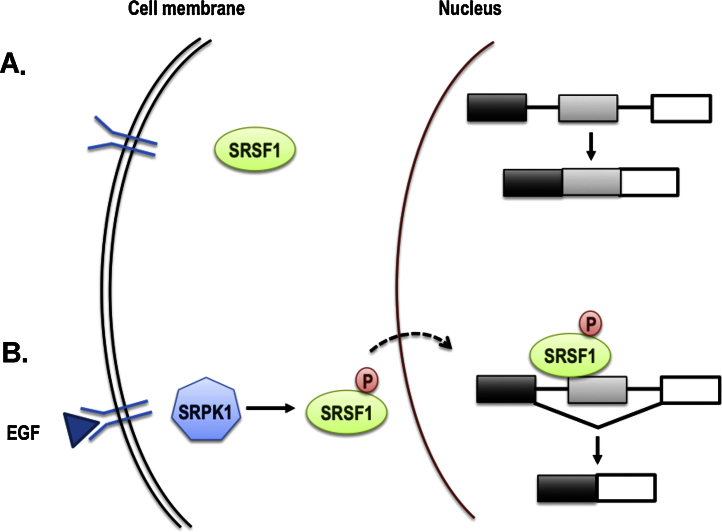
Regulation of alternative splicing by signalling. (A) In unstimulated cells, SR proteins reside in the cytoplasm. (B) Activation of trasmembrane receptors (for example EGF-receptor) stimulates kinases such as SRPK1, which in turn phosphorylate SR proteins; they move into the nucleus to change the splicing pattern of various transcripts. P, denotes phosphorylated state.

Splice factors, similar to transcription factors, are integrated in signalling pathways, such as those regulated by transmembrane receptor activation. Binding of a signalling molecule to its receptor, phosphorylates and thus activates a SF, which then translocates into the nucleus to regulate processing of its target RNAs (Fig. 3**)**.

### 3.3 AS and cancer

Given the extent of AS, it is not surprising that there are thousands of isoforms specifically associated with disease progression, including oncogenesis [22]. Splicing variants are described in almost every class of molecules, including growth factors, tyrosine receptors, tumour suppressors and oncogenes. Many times the splicing isoforms have opposing functions e.g pro- or anti-angiogenic, pro- or anti-apoptotic [see recent reviews [22], [23]]. Two recent reports in Nature highlight the close connection between Myc, one of the most important oncogenes, and the splicing machinery [24], [25]. It is therefore not surprising that AS manipulation has recently emerged as a novel area in which therapeutic intervention may be designed, with the general idea being to try and switch isoforms that are characteristic to cancer and assist in its progression, to their normal counterparts [26].

### 3.4 Modulation of splicing for therapeutic benefit

**Fig. 4:**
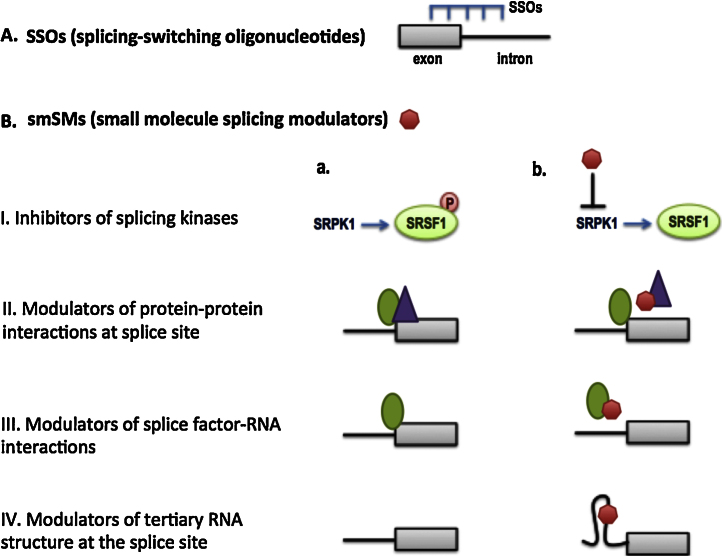
Different mechanisms for potential spliced-based therapeutics. (A) Splice-switching oligonucleotides. (B) Small molecule splicing modulators (red shape) can (i) inhibit activation of splice factors or (ii⿿iv) can modulate selection of splice sites.

One of the greatest advances in the development of splicing therapeutics so far is the concept of splicing-switching oligonucleotides (SSOs) (Fig. 4A). These are complementary sequences designed to bind exon-intron junctions or intronic/exonic regulatory elements and thus affect splicing outcomes.

Another concept, that of small molecules splicing modulators (smSM) that can be used in therapeutics, has also gained the interest of the splicing field recently. Theoretically smSMs may be designed at several levels that can affect splicing outcomes (Fig. 4B), such as inhibitors of kinases that are specific regulators of splice factors (like the example related to SRPK1 from our own work described below), modulators of protein⿿protein or protein-RNA interactions at splice sites or modulators of RNA tertiary structure at splice sites.

For a long time there has been reluctance on whether splicing therapeutics can be specific enough, given the large number of splice sites and their loose consensus sequences. However, the unique characteristics of a splice site are given by many factors including the secondary and tertiary RNA structure, interactions of splice factors bound to those sites either with each other or with RNA. Recently, two studies have screened large chemical libraries for modulators of SMN splicing in a quest to develop novel therapeutics for spinal muscular atrophy [27], [28]. Remarkably, deep sequencing showed that their lead compounds are highly specific (affect less than 10 additional splice sites). Specifically, one of the reports describes that the mechanism of action of one of the compounds is through disruption of the interaction between a splice factor and RNA [28].

## 4 SRPK1 as a novel therapeutic target in PCa

Serine-arginine protein kinase 1 (SRPK1) is a kinase that phosphorylates SR- proteins and modulates their activity. It has been shown to be upregulated in numerous cancers⿿breast, colon andpancreatic carcinomas [29], hepatocellular carcinoma [30], esophageal squamous carcinomas [31], ovarian [32] and lung cancers [33] or glioma [34]. We have recently shown that it is strongly upregulated in PCa tissues and correlates with disease stage and invasion [35].

**Fig. 5:**
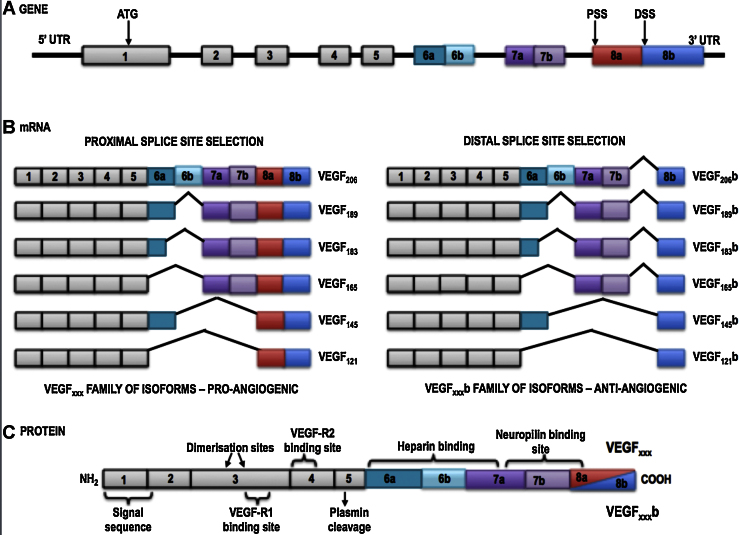
VEGF splice variants. Selection of a distal splice site (DSS) in the terminal exon results in formation of anti-angiogenic ⿿b⿿ isoforms.

We have reported previously [36] that SRPK1 is a key regulator of the balance between two splice isoforms ⿿ VEGF165a, the canonical one that is proangiogenic and VEGF165b, resulting from an alternative 3⿲ splice site in the terminal exon (see Fig. 5) that has been shown in numerous studies to be anti-angiogenic [37], [38]. This is accomplished through phosphorylation of the splice factor SRSF1. Moreover, knockdown of SRPK1 in a colon carcinoma cell line decreased tumour growth and microvessel density [36].

Based on this data we enquired whether this is also true in PCa. Knockdown of SRPK1 switched VEGF splicing towards the antiangiogenic isoform in PC3 cells line and decreased tumour growth in xenografts as well as microvessel density in tumours [39]. Although SRPK1 has been shown to regulate other tumorigenic functions [40], we have not found any evidence that SRPK1 changes proliferation, migration or invasion in PC3 cells. Moreover, in a rescue experiment, we have shown that if VEGF is expressed from a construct that cannot be spliced, it rescues the tumour growth phenotype seen in SRPK1 knockdown cells, therefore suggesting that the effect we see in PC3 is mainly due to affecting angiogenesis through a switch in VEGF splicing. Finally, in a therapeutic proof-of-principle experiment we have shown that intraperitoneal administration of a specific SRPK1 inhibitor (SPHINX) is able to reduce tumour growth in an orthotopic mouse model of PCa.

Our human data on a cohort of 110 patients with PCa showed that SRPK1 expression is strongly associated with disease stage and invasion but not with Gleason score. This supports our findings in the pre-clinical studies that SRPK1 is a determinant of angiogenesis in PCa, as such it would not affect cell morphology (and therefore Gleason score) but contribute to its aggressiveness by stimulating angiogenesis.

## 5 Concluding remarks and future directions

The failure of clinical trials using antiangiogenics in PCa so far, despite strong evidence that angiogenesis is crucial for PCa progression, has pointed out that we need to understand better the biology of vessels and angiogenesis in tumours, the various functions of angiogenic regulators and design better targeted treatments. Once such example might be the inhibition of SRPK1, which is highly expressed in PCa and drives expression of the pro-angiogenic VEGF splice isoforms, and not the beneficial anti-angiogenic ones, which are also inhibited by the current anti-VEGF therapies.

## References

[1] Jayson G.C., Kerbel R., Ellis L.M., Harris A.L. (2016). Antiangiogenic therapy in oncology: current status and future directions. Lancet.

[2] Bottsford-Miller J.N., Coleman R.L., Sood A.K. (2012). Resistance and escape from antiangiogenesis therapy: clinical implications and future strategies. J. Clin. Oncol..

[3] Mukherji D., Temraz S., Wehbe D., Shamseddine A. (2013). Angiogenesis and anti-angiogenic therapy in prostate cancer. Crit. Rev. Oncol. Hematol..

[4] Siegel R.L., Miller K.D., Jemal A. (2015). Cancer statistics, 2015. CA Cancer J. Clin..

[5] Clarke J.M., Armstrong A.J. (2013). Novel therapies for the treatment of advanced prostate cancer. Curr. Treat. Options Oncol..

[6] Karantanos T., Corn P.G., Thompson T.C. (2013). Prostate cancer progression after androgen deprivation therapy: mechanisms of castrate resistance and novel therapeutic approaches. Oncogene.

[7] Drake C.G., Sharma P., Gerritsen W. (2014). Metastatic castration-resistant prostate cancer: new therapies, novel combination strategies and implications for immunotherapy. Oncogene.

[8] Hanahan D., Weinberg R.A. (2011). Hallmarks of cancer: the next generation. Cell.

[9] Duque J.L., Loughlin K.R., Adam R.M., Kantoff P.W., Zurakowski D., Freeman M.R. (1999). Plasma levels of vascular endothelial growth factor are increased in patients with metastatic prostate cancer. Urology.

[10] Bok R.A., Halabi S., Fei D.T., Rodriquez C.R., Hayes D.F., Vogelzang N.J. (2001). Vascular endothelial growth factor and basic fibroblast growth factor urine levels as predictors of outcome in hormone-refractory prostate cancer patients: a cancer and leukemia group B study. Cancer Res..

[11] Weidner N., Carroll P.R., Flax J., Blumenfeld W., Folkman J. (1993). Tumor angiogenesis correlates with metastasis in invasive prostate carcinoma. Am. J. Pathol..

[12] Kelly W.K., Halabi S., Carducci M., George D., Mahoney J.F., Stadler W.M. (2012). Randomized, double-blind, placebo-controlled phase III trial comparing docetaxel and prednisone with or without bevacizumab in men with metastatic castration-resistant prostate cancer: CALGB 90401. J. Clin. Oncol..

[13] Ebos J.M., Lee C.R., Christensen J.G., Mutsaers A.J., Kerbel R.S. (2007). Multiple circulating proangiogenic factors induced by sunitinib malate are tumor-independent and correlate with antitumor efficacy. Proc. Natl. Acad. Sci. U. S. A..

[14] Madan R.A., Karzai F.H., Ning Y.M., Adesunloye B.A., Huang X., Harold N. (2016). Phase II trial of docetaxel, bevacizumab, lenalidomide, and prednisone in patients with metastatic castration-resistant prostate cancer. BJU Int..

[15] Pan Q., Shai O., Lee L.J., Frey B.J., Blencowe B.J. (2008). Deep surveying of alternative splicing complexity in the human transcriptome by high-throughput sequencing. Nat. Genet..

[16] Wang E.T., Sandberg R., Luo S., Khrebtukova I., Zhang L., Mayr C. (2008). Alternative isoform regulation in human tissue transcriptomes. Nature.

[17] de Klerk E., Peter 't Hoen A.C. (2015). Alternative mRNA transcription, processing and translation: insights from RNA sequencing. Trends Genet..

[18] Ule J., Ule A., Spencer J., Williams A., Hu J.S., Cline M. (2005). Nova regulates brain-specific splicing to shape the synapse. Nat. Genet..

[19] Yang J., Hung L.H., Licht T., Kostin S., Looso M., Khrameeva E. (2014). RBM24 is a major regulator of muscle-specific alternative splicing. Dev. Cell.

[20] Warzecha C.C., Jiang P., Amirikian K., Dittmar K.A., Lu H., Shen S. (2010). An ESRP-regulated splicing programme is abrogated during the epithelial-mesenchymal transition. EMBO J..

[21] Warzecha C.C., Sato T.K., Nabet B., Hogenesch J.B., Carstens R.P. (2009). ESRP1 and ESRP2 are epithelial cell-type-specific regulators of FGFR2 splicing. Mol. Cell.

[22] Oltean S., Bates D.O. (2014). Hallmarks of alternative splicing in cancer. Oncogene.

[23] Biamonti G., Catillo M., Pignataro D., Montecucco A., Ghigna C. (2014). The alternative splicing side of cancer. Semin. Cell Dev. Biol..

[24] Koh C.M., Bezzi M., Low D.H., Ang W.X., Teo S.X., Gay F.P. (2015). MYC regulates the core pre-mRNA splicing machinery as an essential step in lymphomagenesis. Nature.

[25] Hsu T.Y., Simon L.M., Neill N.J., Marcotte R., Sayad A., Bland C.S. (2015). The spliceosome is a therapeutic vulnerability in MYC-driven cancer. Nature.

[26] Oltean S. (2015). Modulators of alternative splicing as novel therapeutics in cancer. World J. Clin. Oncol..

[27] Naryshkin N.A., Weetall M., Dakka A., Narasimhan J., Zhao X., Feng Z. (2014). Motor neuron disease: sMN2 splicing modifiers improve motor function and longevity in mice with spinal muscular atrophy. Science.

[28] Palacino J., Swalley S.E., Song C., Cheung A.K., Shu L., Zhang X. (2015). SMN2 splice modulators enhance U1-pre-mRNA association and rescue SMA mice. Nat. Chem. Biol..

[29] Hayes G.M., Carrigan P.E., Miller L.J. (2007). Serine-arginine protein kinase 1 overexpression is associated with tumorigenic imbalance in mitogen-activated protein kinase pathways in breast, colonic, and pancreatic carcinomas. Cancer Res..

[30] Zhou B., Li Y., Deng Q., Wang H., Wang Y., Cai B. (2013). SRPK1 contributes to malignancy of hepatocellular carcinoma through a possible mechanism involving PI3K/Akt. Mol. Cell. Biochem..

[31] Ren G., Sheng L., Liu H., Sun Y., An Y., Li Y. (2015). The crucial role of SRPK1 in TGF-beta-induced proliferation and apoptosis in the esophageal squamous cell carcinomas. Med. Oncol..

[32] Odunsi K., Mhawech-Fauceglia P., Andrews C., Beck A., Amuwo O., Lele S. (2012). Elevated expression of the serine-arginine protein kinase 1 gene in ovarian cancer and its role in Cisplatin cytotoxicity in vitro. PLoS One.

[33] Gout S., Brambilla E., Boudria A., Drissi R., Lantuejoul S., Gazzeri S. (2012). Abnormal expression of the pre-mRNA splicing regulators SRSF1, SRSF2, SRPK1 and SRPK2 in non small cell lung carcinoma. PLoS One.

[34] Wu Q., Chang Y., Zhang L., Zhang Y., Tian T., Feng G. (2013). SRPK1 dissimilarly impacts on the growth, metastasis, chemosensitivity and angiogenesis of glioma in normoxic and hypoxic conditions. J. Cancer.

[35] Bullock N., Potts J., Simpkin A.J., Koupparis A., Harper S.J., Oxley J. (2016). Serine-arginine protein kinase 1 (SRPK1), a determinant of angiogenesis, is upregulated in prostate cancer and correlates with disease stage and invasion. J. Clin. Pathol..

[36] Amin E.M., Oltean S., Hua J., Gammons M.V., Hamdollah-Zadeh M., Welsh G.I. (2011). WT1 mutants reveal SRPK1 to be a downstream angiogenesis target by altering VEGF splicing. Cancer Cell.

[37] Bates D.O., Cui T.G., Doughty J.M., Winkler M., Sugiono M., Shields J.D. (2002). VEGF165b, an inhibitory splice variant of vascular endothelial growth factor, is down-regulated in renal cell carcinoma. Cancer Res..

[38] Harper S.J., Bates D.O. (2008). VEGF-A splicing: the key to anti-angiogenic therapeutics?. Nat. Rev. Cancer.

[39] Mavrou A., Brakspear K., Hamdollah-Zadeh M., Damodaran G., Babaei-Jadidi R., Oxley J. (2015). Serine-arginine protein kinase 1 (SRPK1) inhibition as a potential novel targeted therapeutic strategy in prostate cancer. Oncogene.

[40] van Roosmalen W., Le Devedec S.E., Golani O., Smid M., Pulyakhina I., Timmermans A.M. (2015). Tumor cell migration screen identifies SRPK1 as breast cancer metastasis determinant. J. Clin. Invest..

